# Does Water Quality Matter for Life Quality? A Study of the Impact of Water Quality on Well-being in a Coastal Community

**DOI:** 10.1007/s00267-022-01673-0

**Published:** 2022-06-25

**Authors:** Ruslan Gunko, Lauri Rapeli, Timo Vuorisalo, Matias Scheinin, Patrik Karell

**Affiliations:** 1 Department of Biology, University of Turku, FI-20014 Turku, Finland; 2 Bioeconomy Research Team, Novia University of Applied Sciences, Raseborgsvägen 9, FI-10600 Ekenäs, Finland; 3 Social Science Research Institute, Åbo Akademi University, Turku, Finland; 4 Department of Environmental Protection, Hanko, Finland and Pro Litore Association, Raseborg, Finland; 5 Department of Biology, Lund University, Ecology Building, 223 62 Lund, Sweden

**Keywords:** life satisfaction, local environment, environmental perception, Baltic Sea, eutrophication, community response

## Abstract

Most studies of life quality are concentrated on a country-level scale, while local differences within a country or area are less studied. Thus, the effect of the environment on life quality on a local scale remains understudied and is often represented by one generalized common factor. In this study, we investigated the effect of an objectively measured environmental quality variable and subjective reflections of this (perceptions of environmental quality) in relation to life quality in a coastal community. Hence, we tested the effect of objective and subjective water quality measures using a model, accounting for other traditional variables (e.g., income and health) that predict life quality variations. Our findings indicate that perceptions of the environment are strongly associated with life quality, whereas objectively measured environmental quality is associated with life quality to a lesser extent. Thus, our results suggest that the impact of the environment on life quality is mediated via the way the environment is perceived (psychological effects) and less by the actual conditions of the environment.

## Introduction

Life quality (hereafter referred to as LQ) is an overall characteristic of people’s well-being in all spheres of daily life (Hörnquist 1990; Felce and Perry 1995; Gasper 2010). Its measurement has been under constant development for a long time. However, in the 21^st^ century, it has become key to assessing human life and has started to play an important role in social and political agendas in developed countries (Costanza et al. 2007; Barcaccia et al. 2013). Establishing standards for and determining crucial factors in definitions of a high quality of life are much-disputed processes and are often speculated upon in the media and among researchers (Craglia 2004; McCann 2004; Helliwell et al. 2012).

For the individual, pursuit of happiness and LQ seems only natural. In fact, it could arguably be considered the ultimate goal of life (Layard and Layard 2011). LQ on a personal level is a subjective judgement about life satisfaction, which represents how a person perceives overall life conditions (Diener et al. 2002; Weber et al. 2015) and evaluates his/her quality of life (Diener 2012; Veenhoven, 2014). Life satisfaction relates to the overall life quality situation, considering life circumstances and personal experience from a long-term perspective (Campbell et al. 1976; Haller and Hadler 2006; Veenhoven 2008). In other words, it reflects subjective LQ (hereafter referred to as sLQ). The pathway to life satisfaction differs among individuals and across countries and continents (Böhnke 2008).

An evaluation of LQ requires careful and rational selection of representative indicators, and it cannot be measured by one simple indicator (OECD 2011). The usefulness of these indicators also depend on the level at which the evaluation is done (global, between countries, regional, between individuals). Most commonly LQ is measured using traditional indicators, which reflect sociodemographic, economic, and environmental conditions, as well as political situations (Voukelatou et al. 2021). However, some of these indicators cannot be considered on a local (individual) level as they represent generalized country-scale estimates (e.g., GDP or political situation). Other traditional indicators, such as income, health, education, and living conditions (Hörnquist 1990; Grzeskowiak et al. 2006; Cooper et al. 2013) are more useful independent of the level of evaluation.

In addition to the sociodemographic and economic indicators also the natural environment is an important indicator in LQ measurements (Keles, 2012). Also, here the level of the evaluation dictates which environmental variables are relevant. Previous studies have found that negative changes in environmental conditions have a direct negative impact on LQ on both individual and community level (Rogers et al. 2012). This impact can cause health issues, affect overall life conditions of generations and potentially be the reason for armed conflict (Hopwood et al. 2005). Furthermore, a number of scholars have reported a strong positive linkage between good state of the environment (objective and perceived) and health-related measures of LQ (Lawton 1983; Mariani et al. 2010; Parra et al. 2010). The access to the natural environment has an important recreational value for human well-being by reducing the stress impact (Björk et al. 2008; Lafortezza et al. 2009). Therefore, current knowledge predicts that the state of the environment can influence life quality through mental, psychological, physical or physiological processes (de Hollander et al. 1999; Velarde et al. 2007; Nisbet et al. 2010).

Exactly which of these processes is most important for LQ can be assessed by comparing the effects of objectively and subjectively measured values of an environmental indicator while controlling for other important indicators of LQ (Tveit et al. 2018). Objective measurements correspond to quantitative estimates of the state of the environment and usually require expert knowledge and use of special equipment for measuring it (Perlaviciute and Steg 2018). For example, different types of pollution: air, noise, and water, and effect of biodiversity (Welsch 2006; Gidlöf-Gunnarsson and Öhrström 2007; Keeler et al. 2012; Pecl et al. 2017). Typically, these objective environmental indicators represent country-scale trends and lack a subjective reflection of the state of environment in LQ studies (Streimikiene 2015).

In contrast, subjectively measured environmental indicators reflect perceptions of environmental conditions in the society (Lee 2008; Petrosillo et al. 2013). The way how people perceive the environment is often related to their concerns, feelings, and experience (Bechtel 1997; Gosling and Williams 2010; Steinke et al. 2017; Gunko et al. 2022). At the same time, the socio-economic status of respondents potentially can affect the way people perceive nature (Ibsen and Ballweg 1969; Canter et al. 1992), which is why we may expect interactive effects between the state of the environment and socio-economic status (e.g., health, education, income level) on LQ.

Water quality (hereafter referred to as WQ) often plays a more important role than other reflections of the state of the environment in coastal communities. The ecosystem services provided by coastal waters (provisioning, regulating, and cultural) represent overall-related activities in coastal communities (Barbier et al. 2011; Blythe et al. 2020), which suggests WQ may have both psychological, physiological, and physical impacts on LQ. For this reason, the relationships between observed WQ and LQ in relation to perceived WQ and LQ deserves attention in the concept of life quality, satisfaction, and happiness.

In this study, we aim to assess the effects of the objective state of the environment (water quality) and people’s own evaluations of the state of the environment in relation to reported sLQ. We examine the conjunction between sLQ and an observable environmental indicator, using a unique combination of water quality data and survey data. By combining these data, we are able to geographically pinpoint areas for which we have measures of both water quality and perceived quality, and also a personal LQ assessment (sLQ). With this analysis, we can contribute to an understanding of the role that the environment plays in formation of sLQ by investigating how the actual state of nature and perceptions of environmental conditions affect sLQ on an individual level.

We investigate the importance of WQ for individual sLQ in a coastal community in two dimensions: objectively measured with scientific instruments (hereafter referred to as oWQ) and subjectively assessed by inhabitants (hereafter referred to as sWQ). We make our predictions of causality between sLQ and WQ (i.e., variation in WQ predicts variation in sLQ) according to the framework in OECD Better life index (Durand 2014), although we want to acknowledge that our inference is based on correlations between sLQ and WQ without a formal test of causality. We hypothesize that both measurements reflecting the state of local environment are important, where oWQ would be an important predictor explaining variation in LQ. We predict an even stronger effect of sWQ on sLQ (perception of WQ) since it reflects the respondents’ personal relationships with local nature and it highlights the importance of these for each individual while also considering other life-related factors, including emotions and feelings (“psychological effect”). We also hypothesize that traditional LQ indicators (particularly income) would affect the role of the selected environmental indicator (WQ) in determining sLQ.

## Methods

Our research was conducted in a coastal community in Finland, which has been named the happiest country in the world for four years in a row, 2018-21 (Helliwell et al. 2021). The study was conducted in the city of Raseborg, located in the southwest of Finland on the Baltic Sea coast (Ekenäs archipelago). The population of Raseborg was 27,528 in 2020 (OSF 2020a). The state of the coastal waters is due to tourism of socio-economic importance to the municipality and its citizens. However, according to SYKE (2019), most waters in the Ekenäs archipelago have a “poor” ecological status and it’s related to the eutrophication as the main issue in the Baltic Sea (HELCOM, 2018). This could potentially affect tourism and the life quality of inhabitants.

### Water quality and survey data

To answer the research questions in the study, we used two types of data: objective (oWQ) and subjective (sWQ) water quality data. The oWQ data were collected by sampling visible water quality variables, such as water turbidity and concentrations of fluorescent, dissolved organic matter (hereafter referred to as fDOM), and chlorophyll *a*, along with variables depicting the physical environment, such as temperature and salinity. In this paper, we use fDOM concentration as a proxy for the level of organic loading, in accordance with Nixon (1995). The fDOM measurements were carried out using an automated underway measurement system equipped with optical sensors, along a coastal transect of approximately 300 nautical miles (550 km), covering the Raseborg archipelago. The oWQ variables were collected during mid-October 2019. Based on corresponding data that cover seasonal variation in WQ, October is the most representative time to reflect the overall status of the waters within the study area. The system was installed in a rigid inflatable boat (Brig N610H) with a 0.4-m draft and water intake at a depth of 0.5 m, enabling the system to operate even in very shallow environments. Data were constantly recorded, together with geospatial referencing information, at 5-second (s) intervals by an EXO2 multiparameter sonde and an associated Handheld Unit (Xylem Inc., United States). As a result, we obtained a total of 14,484 observations. Data collection, calibration and handling are described in detail in Scheinin and Asmala (2020). Additionally, each variable was corrected for the statistical analysis using salinity as a way to link observations to the physical characteristics of the environment.

The sWQ data were obtained from a survey conducted among the city’s inhabitants and people who owned or rented a property in Raseborg. The questionnaire had 16 questions (Appendix 1), focusing on people’s assessments of the state of the coastal WQ in areas close to their homes. The respondents were also asked to assess the significance of natural benefits and importance of the state of the environment surrounding them. To answer these questions, the respondents used a 0 to 10 scale, where 0 means very low/poor, and 10 is very high/good. No other scale description was specified, and respondents were free in their responses without any further guidance. This estimation of subjectively perceived water quality has previously been found to correlate strongly with objectively measured fDOM water quality (Gunko et al. 2022). At the same time, we collected sociodemographic parameters such as gender, age, education level, perception of income level (hereafter: income), and perception of health situation (hereafter: health), and asked respondents to describe their relationship with the property (owning/renting), the type of property and how long they had lived there or owned it. At the end of the questionnaire, we asked respondents to express their overall life satisfaction (sLQ) and happiness level using the same 0–10 scale. Additionally, we asked people to assess their emotional attachment to the place (or property) and their opinions about the potential impact on WQ (see translation of the questionnaire in Appendix 1 for question wordings).

**Fig. 1:**
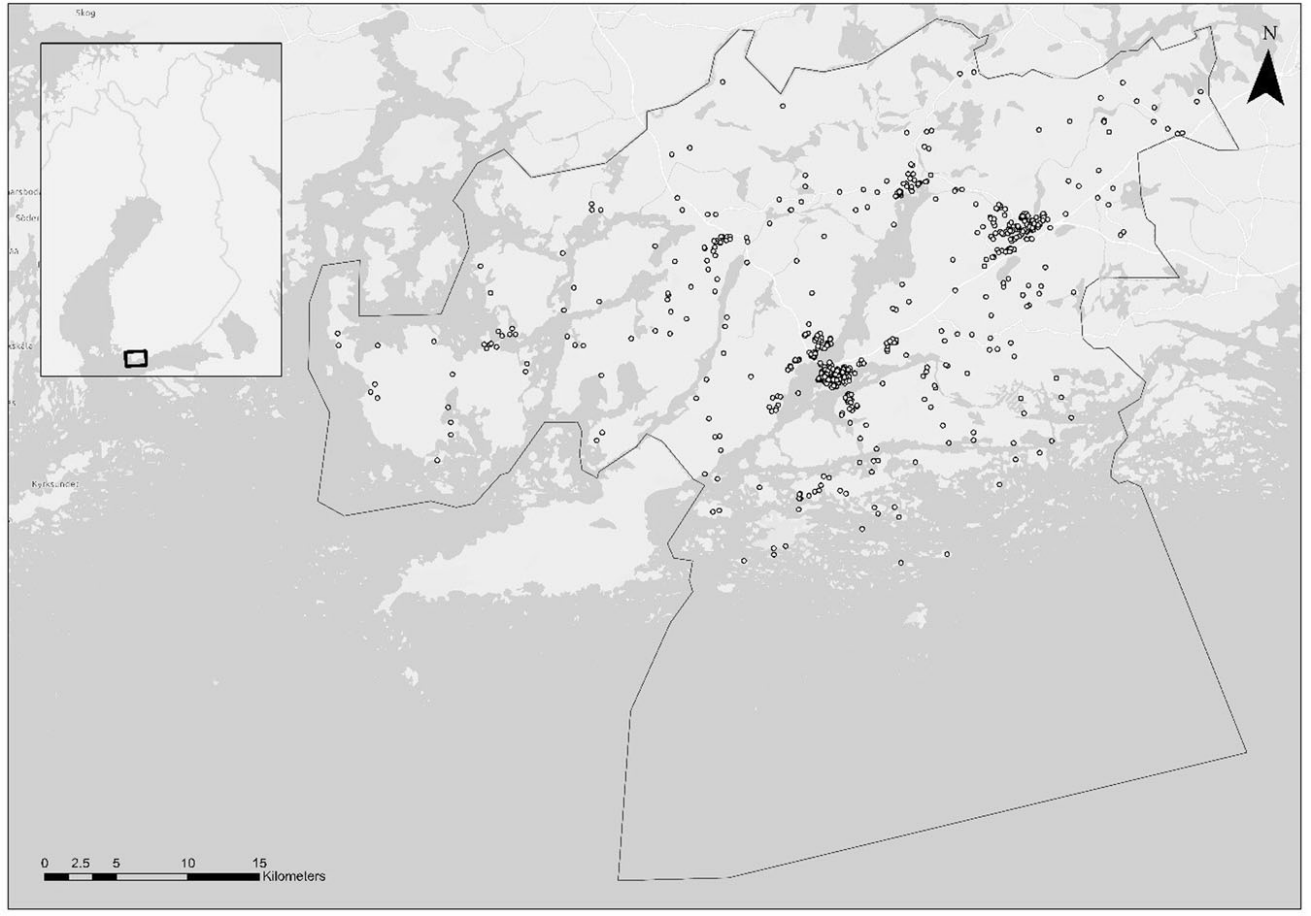
Study area with mapped survey respondents’ locations

All survey answers were georeferenced on the basis of the self-reported address of the respondents so that the survey responses could be linked to an objective measure of WQ in the physical environment. Respondents were recruited by distributing the survey questionnaire during local events, via Facebook advertising in local groups, local newspaper advertising, and by contacting households directly via post-boxes with promotional flyers, which invited respondents to go to a web address and respond to the survey. Potential respondents had the option to answer the survey in the two official languages Finnish and Swedish, as well as in English. After collecting the responses, all answers were sorted, and unclear or unsuitable responses (e.g., incomplete surveys, those with missing address data, or respondents under the age of 16, etc.) were excluded, resulting in 769 complete responses (Fig. 1). Given that a random sample of the target population (=inhabitants of the municipality of Raseborg) was not available, we applied a post-survey weight based on the population age and gender structure in Raseborg (OSF 2020a) to correct for sociodemographic skewness in our sample. The weighted sample, used in the analysis, is representative of the target population in terms of age and gender. Most importantly, the sample includes respondents from all parts of the Raseborg coastline and is representative of the different environmental contexts within the municipality. By including the relevant variation in the environmental conditions, the sample provides a robust perspective into the relationship between (varying) state of the environment and life quality. Additionally, by representing 2,8 % of the target population, the sample is sizable enough for statistical analysis.

The objective data were transferred to ArcMap 10.5 (ESRI 2017) and interpolated using the Diffusion Kernel method, with the land as a barrier. We split the study area into watersheds using a 10-m digital elevation model (NLS 2016) and created, for each watershed, a 100-m offshore buffer. The mean value of oWQ variables was calculated for respective watersheds. Thus, each watershed had a WQ measure package (fDOM and salinity measures). All survey answers were allocated to watersheds (79 watersheds in total) according to their georeferences.

### Statistical analysis

For the statistical analysis, we constructed a variable, oWQ, based on the residuals between fluorescent dissolved matter, fDOM, and surface water salinity. In coastal waters, these variables are strongly linked to one another through the level of freshwater input and the degree of mixing with saline seawater (Asmala et al. 2014). Therefore, lower salinity levels reflect waters which are less exposed to the open sea, and higher salinity reflects higher exposure, which strongly affects the mixing. By using the residuals between fDOM and surface water salinity we aim to simplify the interpretation of oWQ and to make the oWQ models comparable with sWQ models (models 1 and 2 in Table 1). Therefore, an increase in our salinity-corrected oWQ variable indicates an increase in water quality.

**Table 1:** List of linear mixed models used in the study. See text in ‘Statistical analyses’ for details about the variables and model structure and comparison

Model	Response variable	Explanatory variables (fixed effects)	Random effect
1	Life satisfaction	oWQ + health + income + rent/own housing + gender + age + education + natural benefits importance + distance to sea	Watershed
2	Life satisfaction	sWQ + health + income + rent/own housing + gender + age + education + natural benefits importance + distance to sea	Watershed
3	Life satisfaction	sWQ*income + health + rent/own housing + gender + age;+ education + natural benefits importance + distance to sea	Watershed

We derived sWQ assessments and life satisfaction answers from the survey. The life satisfaction variable reflects respondents’ overall perception of LQ (Weber et al. 2015). Our WQ data (both objective and subjective) showed a large variation in measures ranging from poor to good in oWQ, as well as in the assessments respondents ranging from 0 to 10 (sWQ) (for descriptive statistics see Appendix 3).

In our statistical approach we first compared two different models, where the aim was to explain variation in life satisfaction among respondents. In the first model, our measurement of the environment was represented by the oWQ variable, and in the second model it was represented by each respondent’s sWQ assessment. The aim of the analysis was to study the effect of the local environment on life satisfaction and understand the nature of this effect. In other words, we tested two reflections of the environment and investigated which had a stronger effect on life satisfaction: an objectively measured state of the environment or people’s own perception of the environment. Both models additionally had, as explanatory variables, sociodemographic factors (collected by surveying people) which are generally associated with life satisfaction: health (Siahpush et al. 2008); income (Boyce et al. 2010; Cheung and Lucas 2015); education (Cheung and Chan 2009; Salinas-Jiménez et al. 2011); rent/own factor of the house/property (Elsinga and Hoekstra 2005; Diaz-Serrano 2009); the age and gender effect (George et al. 1985; Fodor et al. 2011; Joshanloo 2018); and the importance of natural benefits (Maller et al. 2006; Biedenweg et al. 2017). Additionally, we tested the effect of distance to the sea on life satisfaction, as previous studies have indicated the importance of water accessibility for aesthetic reasons (Wheeler et al. 2012). We then compared the data to determine which of the two WQ estimates (objective or subjective) better explained variations in life satisfaction by a simple comparison of the estimates in the models. We then chose the more competitive model. We compered marginal and conditional R², and the model with higher R² was considered as a more competitive model (Nakagawa and Shielzeth 2013).

In the second step, we investigated whether the putative WQ effect in the more competitive model of life satisfaction would differ depending on the sociodemographic variable of “income level”. We predicted that income level would explain variation in life satisfaction and also expected it to have an impact on how people perceive WQ. To achieve this, we ran the most competitive model (above), including an interaction between WQ and the income explanatory variable. A detailed list of the models and variables used in the study are presented in Table 1. We validated the models graphically (Zuur 2009) and found that model assumptions of homogeneity of variance and normality of residuals were met. All models were run using R statistical software v. 3.6.1. (R Core Team 2014).

## Results

According to the survey answers, the level of life satisfaction in Raseborg is close to the average level in Finland: 88.7% in Raseborg (with respondents evaluating their life satisfaction (sLQ) by giving it a score of 8, 9, or 10 on the 0–10 scale) vs. 91% for the country as a whole (OSF 2020b).

### Effect of the environment on life satisfaction

**Fig. 2:**
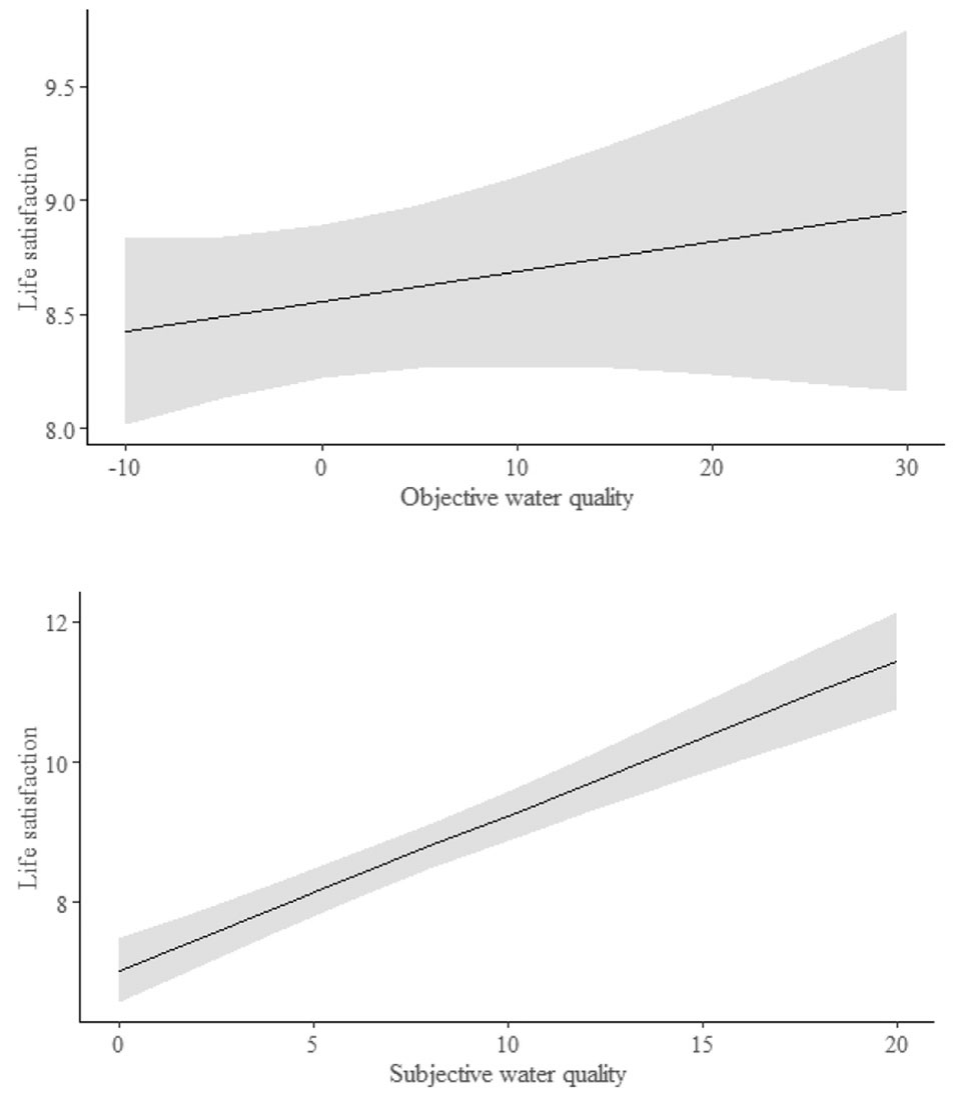
The relationship between life satisfaction (sLQ) and water quality where (a) water quality is objectively measured (residuals between fDOM and salinity), and where (b) water quality is assessed by the respondents. The models take into account income, health, education, age, gender, relationship with property (rent/own) of the respondents, and importance of natural benefits for them (see Table 2 and Table 3 for statistics). The values of life satisfaction and subjective water quality are weighted according to the population structure

We first tested whether oWQ or sWQ better explained variations in life satisfaction (Models 1 vs 2, Table 1). In model 1 we found that our objective measure of WQ did not have a significant impact on sLQ (*t* = 1.088, df = 52.415, *p* = 0.282; Fig. 2a). However, there were clear effects associated with the sociodemographic parameters of health, income, gender, age, education, property ownership, and natural benefits in terms of the importance for sLQ (Table 2). Distance to the sea had no effect on sLQ (Table 2). The overall model fit was as follows: marginal R² = 0.778; conditional R² = 0.779.

**Table 2:** Linear mixed model presenting the relationship between life satisfaction and objective water quality (Model 1). The model includes the effects of sociodemographic variables: income level presented by four groups (1 – living comfortably with present income, 2 – coping, 3 – difficult with present income, 4 – very difficult), health level presented by three groups (1 – higher health level, 2 – intermediate health level, 3 – lower health level), level of education presented by two groups (1 – lower education level, 2 – higher education level), rent/own presented by three groups (1 – own, 2 – rent, 3 other) and gender presented by two groups (1 – females, 2 – males)

Variable	Estimate ± SE	DF	*t*	*P*
oWQ (residuals for fDOM and salinity)	0.013 ± 0.012	52.415	1.088	0.282
Health: intermediate health level	−0.340 ± 0.148	737.363	−2.304	<0.05
Health: lower health level	−1.731 ± 0.301	740.735	−5.761	<0.001
Income: coping on present income	−0.427 ± 0.147	740.968	−2.904	<0.01
Income: difficult on present income	−0.921 ± 0.213	739.936	−4.317	<0.001
Income: very difficult on present income	−0.824 ± 0.372	740.736	−2.218	<0.05
Rent/own: rent property	0.259 ± 0.193	715.708	1.344	0.179
Rent/own: other property status	0.725 ± 0.226	740.674	3.207	<0.01
Gender: males	0.597 ± 0.141	740.942	4.251	<0.001
Age	0.032 ± 0.005	678.636	6.816	<0.001
Education: higher education level	−0.334 ± 0.135	740.948	−2.467	<0.05
Natural benefits importance	0.764 ± 0.020	740.848	37.932	<0.001
Distance to sea	0.055 ± 0.046	14.062	1.198	0.251

In the second model (Model 2), we replaced oWQ with respondents’ sWQ assessment (marginal R² = 0.800; conditional R² = 0.801). In this more competitive model, we found a strong relationship between sWQ and sLQ (*t* = 9.151, df = 736.011, *p* < 0.001; Fig. 2b). Besides the subjective water quality effect, the analysis showed a strong effect of sociodemographic factors on sLQ (Table 3; see also Table 2 for comparable estimates). Health (a three-level factor) showed a strong negative effect, with respondents with low health levels reporting the lowest levels of sLQ and respondents with intermediate health levels reporting intermediate levels of sLQ (Table 3: health). Income level (a four-level factor) had a strong impact on sLQ, with respondents with higher income levels being more satisfied with life (Table 3: income). In contrast to our prediction, we found that education had a significant negative effect on sLQ, with people educated to a higher level giving lower scores of sLQ (Table 3: education). Property ownership (the rent/own factor) showed that renting vs. ownership did not affect sLQ, whereas people who described their relationships with properties as “other” (i.e., people who described their property as “commercial”; see Appendix 1) were more satisfied with life (Table 3: satisfaction levels for rent/own and “other”). We found a strong positive effect of gender on sLQ, with males being more satisfied than females. Age had a positive effect on sLQ, where sLQ scores increased with the age of respondents. Respondents who perceived natural benefits as being important were more satisfied with life (Table 3: natural benefits importance). The distance to the sea did not have an effect on respondents’ sLQ (Table 3: distance to the sea).

**Table 3:** Linear mixed model of the relationship between life satisfaction and subjective water quality assessment (Model 2). The model includes the same socio-demographic variables as Table 2

Variable	Estimate ± SE	DF	*t*	*P*
sWQ	0.221 ± 0.024	736.011	9.151	<0.001
Health: intermediate health level	−0.355 ± 0.140	740.115	−2.531	<0.05
Health: lower health level	−1.546 ± 0.285	739.215	−5.416	<0.001
Income: coping on present income	−0.414 ± 0.139	740.990	−2.969	<0.01
Income: difficult on present income	−0.861 ± 0.202	740.921	−4.255	<0.001
Income: very difficult on present income	−0.890 ± 0.352	740.561	−2.525	<0.05
Rent/own: rent property	0.209 ± 0.183	707.074	1.146	0.252
Rent/own: other relationship with property	0.632 ± 0.215	740.763	2.944	<0.01
Gender: males	0.446 ± 0.134	739.795	3.322	<0.001
Age	0.031 ± 0.004	698.034	6.945	<0.001
Education: higher education level	−0.214 ± 0.129	740.842	−1.662	<0.05
Natural benefits importance	0.613 ± 0.025	740.375	24.217	<0.001
Distance to sea	0.054 ± 0.045	17.690	1.185	0.251

### Can socioeconomic factors affect the role of environmental quality in LQ assessment?

**Fig. 3:**
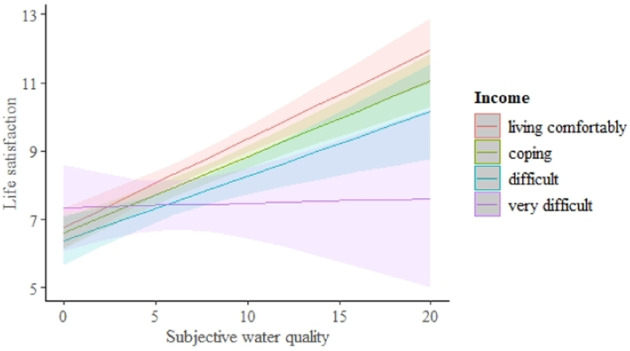
The interaction between income level and water quality assessed by the respondents, and its effect on life satisfaction (see Table 2 for statistics). The values of life satisfaction and subjective water quality are weighted according to the population structure

In model 3 we added an interaction between income level and subjective water quality (income * sWQ) to the life satisfaction model with sWQ (Model 2) in order to assess whether the effect of water quality on life satisfaction could be socio-economy-dependent. We found that subjectively assessed WQ did not affect the sLQ of respondents with a very low income level (indicated by the response “very difficult on present income”), whereas this relationship was evident for those respondents with a higher income level (very low income: *t* = −2.620, df = 727.905, *p* < 0.01; Fig. 3; see Appendix 2 for detailed statistics relating to the complete model). The overall model fit was as follows: marginal R² = 0.801; conditional R² = 0.803.

## Discussion

Determining the factors that affect life quality (LQ) is a complex process, requiring an interdisciplinary approach and analysis of a range of variables. Relationships between life quality and the state of the environment are typically studied on larger scales and do not include local features. Moreover, analyses incorporating a subjective reflection of the environment are often generalized and rely on rough estimates (e.g., OECD 2011). In this study, we aimed to investigate the effect of the environment on the inhabitants own perception of LQ (sLQ) on a local scale (life satisfaction). We tested the impact of both objective measurements of the state of the environment (oWQ) and corresponding reflections by local people, indicating their perceptions of the state of the environment (sWQ). Our statistical inference is correlative and based on the assumption that the WQ estimates explain variation in subjective LQ due to its impact on well-being and mental health (sensu Durand 2014). We conducted our research in a coastal area, and therefore used WQ as the main indicator of the environmental factor for LQ measures. For coastal communities, WQ is a vital aspect of environmental health and something that locals are likely to pay attention to. According to the latest HELCOM report (2018), WQ is a major concern in the Baltic Sea due to the fact that more than 97% of the coastal waters are suffering from eutrophication.

Our findings strongly indicated that the perceived psychological experience of the environment is important in sLQ evaluation. In contrast, the objective state of the environment did not have an equally strong effect on people’s sLQ. However, this does not reduce the importance of the actual state of the environment, but rather, it highlights the significance of perceptions of the state of the environment in the relationship between sLQ and the environment. In other words, our results suggest that subjective perceptions of the environment should be taken into account in LQ evaluations.

Involving societies in consultations and reviews of management plans is one of the cornerstones of the EU Water Framework Directive (European Commission 2000). The opportunity to have an impact on water management projects (related to groundwater, inland and coastal waters across the EU) and to critically review these is granted to the public by The Aarhus Convention (Ebbesson et al. 2014). Thus, the importance of sWQ data for successful management of the environment is indisputable. However, public opinions collected through surveys can present potential challenges related to cognitive problems, attitude effects and social desirability (Bertrand and Mullainathan 2001). Despite the potential challenges, the accuracy of sWQ data when compared with oWQ data on a local level encourages us about its reliability (Gunko et al. 2022). Moreover, the weak, or often, lack of consideration of subjective environmental indicators (or ignorance of the importance of local-scale evaluations of these) in LQ studies (Petrosillo et al. 2013; Streimikiene 2015) emphasizes how incomplete our understanding is of the effects of perceptions of environmental factors on LQ variations within countries. Previous studies have often generalized subjective reflections of the environment to a country-wide level or even ignored them due to the global scale of the assessment. By comparing the effect of oWQ versus the effect of sWQ on sLQ we here show that LQ variation on a local level can vary substantially depending on the perceived quality of the local environment.

Perceptions of the environment or self-interpretation of visual information is based on an understanding of the visible environment and is dependent on a person’s experiences (Renn et al. 1992). The strong positive effect of age on sLQ found in our study supports previous findings.

By juxtaposing objective and subjective data, we have previously found using the same approach as here that the general public is typically able to adequately evaluate the state of the environment (Gunko et al. 2022). However, despite the high accuracy level of sWQ in relation to oWQ (Gunko et al. 2022), we here find that the sWQ measure is a better predictor of people’s estimation of their LQ than oWQ. This is a novel finding, which highlights the ‘psychological effect’ of the environment on sLQ. There is a number of scholars highlighting positive associations between nature and human physical and mental health (Kamitsis and Francis 2013; Ideno et al. 2017; White et al. 2019; Martin et al. 2020), which indicates that the environment impacts health and well-being and thereby LQ. Furthermore, in the current study, we evaluated the hypothesis that the effect of the environment on sLQ is conditional on the income level (Model 3). We find that the environmental quality is a less important determinant of LQ for people with lower income level (Fig. 3). Our interpretation of this result is that respondents with lower income levels prioritise other socio-economic aspects of life quality higher, and that the environment is therefore less important. As the income level increases and thereby sLQ improves, also the state of the living environment starts to play a more important role. Our findings are consistent with the results of Ferreira and Moro (2013) who found that the effect of water pollution on wellbeing depended on income. Therefore, we interpret our finding such that economic security (income level) predominates over environmental factors (sWQ) in determining LQ. However, we found no overall effect of income level on sLQ, which can be explained by a higher proportion of pensioners (65 years and older) in the population structure of Raseborg, compared to the country average among Finnish municipalities (26.7% against 21.8% in 2018, and 27.2% against 22.3 in 2019) (OSF 2020a). The lower importance of income level for older cohorts could reflect lower levels of family responsibility and associated pressures (Cheung and Lucas 2015).

The weak effect of oWQ on sLQ could be misinterpreted and promptly misdirect the discussion of the possibility that the actual state of the environment is not important for LQ. We would argue that this is not the case but rather that we need to keep in mind potential mismatches in perceptions of the environment and our understanding of direct and indirect impacts of the actual state of the environment on sLQ. In other words, inhabitants could make emotionally driven overestimations of environmental quality, which makes subjective perceptions of the environment more important indicators of LQ than objective measurements. We can also not rule out that the public to some extent use other cues to assess water quality (other type of pollution or garbage). Nevertheless, we believe this is unlikely since eutrophication is the foremost concern of both authorities and the public regarding the Baltic Sea (HELCOM 2018). Thus, both objectively and subjectively measured environmental indicators should be used in LQ evaluations, especially in the case of global-scale assessments, when objective LQ indicators like GDP often prevail over environmental indicators. Solely using perceptions of the environment can potentially lead to an overestimation of the role of environmental quality (Betti et al. 2020) and future studies should consider additional measures of both oWQ and sWQ, as well as well-being, to assess the drivers of the mismatch in their predictability of life satisfaction.

An additional challenge in the use of environmental indicators for LQ assessment is often the scale of the study and a failure to consider differences between environmental conditions within countries or larger areas (e.g., neighbouring countries). More studies are needed which investigate the local scale and analyse detailed environmental factors (Rehdanz and Maddison 2005). The role of local governance is increasing as administrative decisions have a direct impact on objective and subjective environmental indicators (Wang et al. 2014). Local environment policies developed by councils (and implementation of these) have an impact on the overall state of the environment and are reflected in citizens’ perceptions of the environment (Phillimore and Moffatt 2004). Namely, the success of local governments in dealing with local environment issues or personal evaluations of these activities by citizens (voters) can affect the values assigned to subjective environmental indicators.

There are potential limitations to the approach of using a single variable to measure LQ and its subjective characteristic. However, a number of scholars have highlighted the advantage of using single-variable use of perceived LQ due to easier collection of the data (Rässler and Riphahn 2006) or clarity for respondents in different parts of the world to understand and reflect their general psychological and health conditions (Diener and Tov 2006; Jylhä 2009; Baćak, V., and Ólafsdóttir 2017). At the same time, usage of subjectively measured LQ has potential challenge in comparison of values on a larger scales, among countries, and subjective measurement quality potentially can be affected by political situation, family issues and age (Helliwell 2008; OECD 2011). These limitations can play a significant role for larger-scale studies and cumulative usage of objective and subjective components is recommended (Brown et al. 1989). This is not the problem in our study due the small scale and the absence of any serious issues on a local scale. Additionally, the advantages of the single-use measurement are easiness in data collection and ability to use the data and results for cross-regions comparison within the country scale.

## Conclusions

Localization of environmental indicators (both subjective and objective) or bottom-up collection of environmental data for LQ studies has the potential to reinforce environmental indicators and embrace more features of the environment in studies of life quality variation. Our findings suggest that the state of the environment is an important factor for locals and their sLQ. The significance of subjective assessments of the local environment (over the objective state of the environment) for LQ demonstrates how local communities can be sensitive to even smaller changes in the natural environment. Thereby, the effects of e.g., global climate change or biodiversity loss will be felt in local communities and are likely to significantly affect the sLQ of residents. Our finding concerning the significant effect of sWQ demonstrates that people are concerned about the local environment and consider their relationship with nature to be among the things that have a significant impact on their LQ.

## Supplementary Information

Appendix_Gunko_1

Appendix_Gunko_2

Appendix_Gunko_3
