# Supplementary material for: Does Water Quality Matter for Life Quality? A Study of the Impact of Water Quality on Well-being in a Coastal Community
Source: Environ Manage. 2022 Jun 25;70(3):464–74. doi: 10.1007/s00267-022-01673-0 (PMC9381611; doi:10.1007/s00267-022-01673-0)
Supplement: Supplementary file 1 — Appendix_Gunko_1 [file 267_2022_1673_MOESM1_ESM.docx]

Thank you for agreeing to take part in this important survey measuring quality of life and state of the environment in Raseborg. In this questionnaire we will ask questions regarding the state of environment around your home or summer cottage and your relation to that environment. This questionnaire only **takes 2-3 minutes** to complete. The study is part of the research conducted in NAME OF UNIVERSITY and the results of the study will be used in my doctoral thesis at the NAME OF UNIVERSITY. Your answers and all related information will be kept confidentially.

By filling out the survey you can join the lottery and win 50€ S-Group gift card. Please, fill out the survey carefully, and if you wish to participate in the lottery, add your contact information.

On behalf of the research team,

In order to combine your answers with actual data from the natural environment (for example, quality of water), please indicate the address of your home OR your summer cottage OR your property. When filling out the survey, please always think about your home or cottage in this address. Note that the information about your address will not at any point in the study be combined with your name or your responses, because the survey is anonymous.

Address:

1. What year were you born?

2. Are you a) female b) male c) prefer not to say

3. What is the highest level of education you have completed? a) primary education; b) high school; c) vocational school d) polytechnic university e) university f) other

4. Which of the following comes closest to how you feel about your household's income nowadays? a) living comfortably on present income b) coping on present income c) difficult on present income d) very difficult on present income

5. How is your health in general? Would you say it is…a) very good b) good c) fair d) bad e) very bad

6. Do you own or rent the property? a) I own it myself or with other people b) I’m renting it c)other

7. Which of the following best describes your relationship with the property? a) permanent home, b) summer cottage or vacation home, c) other (specify)

8. How many years have you lived in this home or summer cottage? a) max 2 years; b) 2 – 5 years; c) 5 – 15 years; d) more than 15 years

9. How emotionally attached do you feel to your property? Please choose a number from 0 to 10, where 0 means not at all emotionally attached and 10 means very emotionally attached.

10. How would you assess the state of the natural environment in your property and in its immediate surroundings? Please choose a number from 0 to 10, where 0 means it is very bad and 10 means it is excellent.

11. How would you assess the quality of the water on your property and in its immediate surroundings? Please choose a number from 0 to 10, where 0 means it is very bad and 10 means it is excellent.

12. In your assessment, what would you say has the biggest impact on the water quality on and around your property? a) industry, b) forestry, c) agriculture, d) other (specify)

13. Some of the benefits one can receive from nature include, for example, spending time in the forest, hiking, swimming, sailing or picking berries. In your assessment, how important are such things for you? Please choose a number from 0 to 10, where 0 means they are not at all important and 10 means they are very important.

14. In your assessment, how important is the state of the environment around your property for your overall wellbeing? Please choose a number from 0 to 10, where 0 means it does not matter at all and 10 means it is extremely important.

15. All things considered, how satisfied would you say you are with your life these days?

Please tell me on a scale from 0 to 10, where 0 means very dissatisfied and 10 means very satisfied.

16. Taking all things together, on a scale of 0 to 10, how happy would you say you are?

Here 0 means you are very unhappy and 10 means you are very happy.

I would like to participate in the lottery: a) yes, b) no

Contact information for the lottery:

Good luck!
