# Supplementary material for: Does Water Quality Matter for Life Quality? A Study of the Impact of Water Quality on Well-being in a Coastal Community
Source: Environ Manage. 2022 Jun 25;70(3):464–74. doi: 10.1007/s00267-022-01673-0 (PMC9381611; doi:10.1007/s00267-022-01673-0)
Supplement: Supplementary file 2 — Appendix_Gunko_2 [file 267_2022_1673_MOESM2_ESM.docx]

| Variable | Estimate ± SE | DF | t | P |
| --- | --- | --- | --- | --- |
| sWQ | 0.258 ± 0.035 | 737.641 | 7.375 | <0.001 |
| Health: intermediate health level | -0.356 ± 0.140 | 737.471 | -2.547 | <0.05 |
| Health: lower health level | -1.610 ± 0.286 | 735.538 | -5.621 | <0.001 |
| Income: coping on present income | -0.156 ± 0.298 | 737.414 | -0.524 | 0.601 |
| Income: difficult on present income | -0.387 ± 0.412 | 736.616 | -0.940 | 0.347 |
| Income: very difficult on present income | 0.576 ± 0.653 | 728.326 | 0.882 | 0.378 |
| Rent/own: rent property | 0.196 ± 0.182 | 707.001 | 1.075 | 0.283 |
| Rent/own: other relationship with property | 0.607 ± 0.214 | 737.843 | 2.832 | <0.01 |
| Gender: males | 0.433 ± 0.134 | 736.889 | 3.230 | <0.01 |
| Age | 0.031 ± 0.004 | 698.060 | 6.896 | <0.001 |
| Education: higher education level | -0.204 ± 0.129 | 737.812 | -1.580 | 0.115 |
| Natural benefits importance | 0.612 ± 0.025 | 737.480 | 24.268 | <0.001 |
| Distance to sea | 0.059 ± 0.045 | 18.436 | 1.304 | 0.208 |
| Income: coping (by sWQ) | -0.036 ± 0.037 | 737.322 | -0.968 | 0.334 |
| Income: difficult (by sWQ) | -0.069 ± 0.055 | 736.561 | -1.265 | 0.206 |
| Income: very difficult (by sWQ) | -0.245 ± 0.094 | 727.905 | -2.620 | <0.01 |

*Appendix 2. The interaction between income and subjective water quality assessment, and its relationship with life satisfaction. The model included the same variables as in Table 2 but with an additional interaction: “income by sWQ”.*
