# Supplementary material for: Does Water Quality Matter for Life Quality? A Study of the Impact of Water Quality on Well-being in a Coastal Community
Source: Environ Manage. 2022 Jun 25;70(3):464–74. doi: 10.1007/s00267-022-01673-0 (PMC9381611; doi:10.1007/s00267-022-01673-0)
Supplement: Supplementary file 3 — Appendix_Gunko_3 [file 267_2022_1673_MOESM3_ESM.docx]

| **Variables** | **N** | **Mean** | | **SD** | **Min** | **Max** |
| --- | --- | --- | --- | --- | --- | --- |
| Satisfaction | 769 | 8.14 | | 1.62 | 0 | 10 |
| sWQ | 769 | 6.78 | | 2.20 | 0 | 10 |
| Natural benefits importance | 769 | 8.92 | | 1.59 | 0 | 10 |
| Age | 769 | 50.35 | | 16.03 | 16 | 83 |
| Distance to sea (m) | 769 | 2250.33 | | 3000.48 | 0.66 | 15415.25 |
|  | | | | | | |
| **Variable** | **N** | | **Proportion (%)** | | | |
| Health: higher health level | 504 | | 65.54 | | | |
| Health: intermediate health level | 224 | | 29.13 | | | |
| Health: lower health level | 41 | | 5.33 | | | |
| Income: living comfortably on present income | 258 | | 33.55 | | | |
| Income: coping on present income | 371 | | 48.24 | | | |
| Income: difficult on present income | 109 | | 14.17 | | | |
| Income: very difficult on present income | 31 | | 4.04 | | | |
| Rent/own: own property | 576 | | 74.91 | | | |
| Rent/own: rent property | 112 | | 14.56 | | | |
| Rent/own: other property status | 81 | | 10.53 | | | |
| Gender: females | 462 | | 60.55 | | | |
| Gender: males | 301 | | 39.45 | | | |
| Education: higher education level | 353 | | 45.91 | | | |
| Education: lower education level | 416 | | 54.09 | | | |

| **Variables** | **N** | **Mean** | **SD** | **Min** | **Max** |
| --- | --- | --- | --- | --- | --- |
| oWQ | 769 | 0.01 | 5.61 | -5.82 | 29.72 |

*Appendix 3. Descriptive statistics of variables collected through survey and oWQ measurements. Proportion column represents proportions of answers per category.*
